# The *LncRNA H19* rs217727 Polymorphism Is Associated with Oral Squamous Cell Carcinoma Susceptibility in Iranian Population

**DOI:** 10.1155/2020/1634252

**Published:** 2020-03-31

**Authors:** Jannan Ghapanchi, Zahra Ranjbar, Mohammad Javad Mokhtari, Fatemeh Koohpeima, Maryam Derakhshan, Bijan Khademi, Hamid Ghaderi, Shamsoddinali Sheikhbahaei, Ehsan Aliabadi

**Affiliations:** ^1^Department of Oral Medicine, School of Dentistry, Shiraz University of Medical Sciences, Shiraz, Iran; ^2^Young Researchers and Elite Club, Department of Biology, Zarghan Branch, Islamic Azad University, Zarghan, Iran; ^3^Department of Operative Dentistry, School of Dentistry, Shiraz University of Medical Sciences, Shiraz, Iran; ^4^Cancer Immunology Group, Shiraz Institute for Cancer Research, School of Medicine, Shiraz University of Medical Sciences, Shiraz, Iran; ^5^DMD Dentist, Imam Reza Clinic, Shiraz, Iran; ^6^Undergraduate Student, Student Research Committee, School of Dentistry, Shiraz University of Medical Sciences, Shiraz, Iran; ^7^Department of Oral and Maxillofacial Surgery, School of Dentistry, Shiraz University of Medical Sciences, Shiraz, Iran

## Abstract

Lack of protein-coding capacity is a main characteristic of long noncoding RNAs (lncRNAs) which, as molecular biomarkers, have found a novel pharmacological application in cancer and are reported to be important regulators of gene expression. *H19* is reportedly involved in cancer progression and tumorigenesis. One of the most common types of head and neck cancers is oral squamous cell carcinoma (OSCC). The main objective of the present study was to evaluate the correlation of OSCC susceptibility with *H19* gene in an Iranian population. This research was performed on 400 subjects of both sexes referred to the Namazi Hospital affiliated with the Shiraz University of Medical Sciences (SUMS). Individuals aged 15-88 years were divided into two groups: pathologically diagnosed patients with new-onset OSCC and healthy controls. After written and informed consent was obtained from the individuals, genomic DNA was extracted. The tetra-primer ARMS-PCR technique was performed for DNA genotyping by the use of specific primer pairs. The susceptibility of OSCC and H19 gene polymorphism sites was further analyzed (rs217727 and rs2107425). The allele and genotype frequencies of *H19* rs2107425 polymorphism were similar between OSCC cases and controls. The *H19* rs217727T allele frequency was significantly higher in OSCC cases (*P* = 0.002), and the polymorphism of *H19* rs217727 was associated with OSCC susceptibility in the codominant (OR = 6.04, 95%CI = 1.70 − 21.42, *P* = 0.001 for TT genotype), dominant (OR = 1.62, 95%CI = 1.08 − 2.43, *P* = 0.01), and recessive (OR = 5.32, 95%CI = 1.51 − 18.69, *P* = 0.003) models. This study showed that rs217727 and OSCC susceptibility were statistically correlated in the Iranian population.

## 1. Introduction

Approximately 8% of malignant tumors among adults belong to the OSCC of head and neck [1]. The oropharynx and the mucous membranes of the mouth are considered as the main origins of more than 90% of squamous cell carcinomas [2]. Individuals exposed to the same environment are highly susceptible to head and neck carcinoma, hence the conclusion that genetic factors play an important role in the occurrence of head and neck carcinoma.

Cell differentiation, gene regulation, chromatin remodeling, cancer cell invasion, and metastasis are considered as the major roles played by lncRNAs. Deregulation of lncRNA is further considered as a major causative factor of numerous diseases in mammals. Different studies have demonstrated the importance of lncRNAs as biomarkers in the diagnosis and prognosis of cancers [3].

*LncRNA H19* is the first discovered lncRNA with its gene, located on human chromosome 11p15.5, encoding a 2.3 kb long, polyadenylated, spliced, and capped noncoding RNA [4]. *LncRNA H19* is transcribed only from maternally inherited alleles [5] and is responsible for controlling genome expression at different levels. *H19* influences the chromatin organization by the recruitment of chromatin modifying complex PRC2, and as a miRNA, it decoys sequestering miR-let7 and miR-106a on posttranscriptional control or as a precursor for miR-675-3p and miR-675-5p. *H19* is further capable of inactivating tumor suppressor proteins through interaction with p53 [6]. Possessing the functions of both oncogenes and suppressor genes [7, 8], *H19* polymorphism has been reported, by many different studies, to be associated with the development and occurrence of tumors [9].

Based on different studies, single-nucleotide polymorphisms (SNPs) are very important markers linking phenotypic changes to DNA-sequence variations. Research in this field is expected to promote the analysis of human system physiology and explain the molecular basis of the diseases. The risk of developing cancer was shown to possibly increase by rs2839698 [10] and decrease by rs2839698 [11] in *H19*. The purpose of the present study was to determine whether OSCC in the Iranian population is related to single-nucleotide polymorphisms (SNPs) in the *lncRNA H19* gene.

## 2. Materials and Methods

### 2.1. Study Subjects

This case-control study comprised 200 patients, diagnosed with new-onset OSCC by two independent pathologists, and 200 healthy individuals without a history of cancer. The entire case group (OSCC) was selected, without any limitations in age and gender, from the Namazi Hospital affiliated with the Shiraz University of Medical Sciences. Healthy people were randomly selected from those referred to Namazi Hospital as controls. As shown in Table 1, a total of 200 patients with OSCC (44 females (22%) and 156 males, (78%)) aged 15-88 years (the mean age and standard deviation were 59.69 ± 16.24) and 200 healthy controls were enrolled in this study. The controls were frequency-matched with the cases on the basis of gender and age (±5 years old). The examined subjects aged 18-88 years. This study was approved by the Ethics Committee of the Shiraz University of Medical Sciences, Iran. The individuals voluntarily agreed to participate in this experiment as a part of a large prospective research project and completed the written informed consent form. The inclusion and exclusion criteria are listed in Table 2.

### 2.2. SNP Genotyping

In this research, the salting out method was employed to isolate DNA from the whole blood samples and DNA purity was measured by spectrophotometry through the use of Eppendorf Biophotometer (Germany). The *LncRNA H19* genomic sequence was obtained from the NCBI site (http://www.ncbi.nlm.nih.gov). The primers were designed for T-ARMS-PCR, a rapid and simple technique for identifying SNPs which were further detected [12]. The location of SNPs and T-ARMS-PCR primers are shown in Table 3. PCR reactions consisted of a total volume of 20 *μ*L, containing 50 ng of genomic DNA, 0.5 *μ*M of each primer, 1 U Taq of DNA polymerase, 250 *μ*M of dNTPs, and 1.5 mM of MgCl_2_. The cycling conditions of PCR included initial denaturation at 95°C for 5 minutes, followed by 30 cycles for rs217727 and rs2107425 at 95°C for 30 seconds, annealing temperature for 30 seconds at 65°C for rs217727, 30 seconds at 65.8°C for rs2107425, and extension temperature for 40 seconds at 72°C, with a final extension of 72°C for 10 minutes. The PCR products were verified by 2% agarose gels. To ensure genotyping quality, approximately 20% of the random samples were sequenced and showed no genotyping error.

### 2.3. Statistical Analysis

The Hardy–Weinberg equilibrium (HWE) was assessed using chi-squared (*χ*^2^) test to compare the expected and observed genotype frequencies among the controls. Further used were the unconditional logistic regression analyses to test the relationship of each SNP with case/control status under different genetic models such as dominant, codominant, recessive, and overdominant. Odds ratio (OR) and its corresponding 95% confidence interval (95% CI) were scored to assess the strength of association between the risk of OSCC and *H19* polymorphisms. The results were considered statistically significant at *P* < 0.05. The analyses were performed using the version 19.0 of the SPSS software.

## 3. Results

The PCR product sizes of *H19* rs217727 polymorphism were 248 bp for C allele, 200 bp for T allele, and 397 bp for internal control; regarding the *H19* rs2107425 polymorphism, the PCR product sizes were 195 bp for T allele, 124 bp for C allele, and 266 bp for internal control at 2% agarose gel.

The frequency of the two SNPs genotypes and their correlations with OSCC risk are given in Table 4. No significant deviations from Hardy–Weinberg equilibrium were observed with regards to the two polymorphisms in the case and control groups (*P* > 0.05). Although the frequency of rs217727C allele was significantly lower in OSCC cases compared to the controls, no significant difference was found between the two groups concerning the rs2107425T allele frequency of *H19* gene (*P* > 0.05), hence the association between the rs217727C allele and the risk reduction of OSCC (*P* = 0.002).

There was no relationship between the OSCC and *H19* rs2107425 genotype. Furthermore, the frequency of TT genotypes of *H19* rs217727 was significantly higher than that in OSCC cases compared to the controls (7.5 vs. 1.5%); TT genotype was associated with 6.04-fold higher risk of OSCC in the codominant model (OR = 6.04, 95%CI = 1.70 − 21.42, *P* = 0.001). In the dominant model, the CT+TT genotypes were associated with 1.62-fold higher risk of OSCC (OR = 1.62, 95%CI = 1.08 − 2.43, *P* = 0.01). Moreover, the *H19* rs217727 polymorphism was related to the 5.32-fold increase in the risk of OSCC in the recessive model (TT vs. CC+CT genotypes) (Table 5).

## 4. Discussion

In the present molecular epidemiological research, the effects of two *H19* gene polymorphisms (rs217727 and rs2107425) on OSCC susceptibility were investigated through a case-control investigation on southwest Iranian subjects. As far as the authors of the present research are concerned, the present is the first research to evaluate the risk of OSCC associated with *H19* polymorphisms in an Iranian population. In the present study, we used multiple inheritance models (codominant, dominant, recessive, and overdominant) to evaluate the associations between SNPs in H19 gene and OSCC risk. Although a significant association was observed between OSCC risk and *H19* rs217727 polymorphism in dominant, codominant, and recessive models, risk of OSCC and *H19* rs2107425 polymorphism were not correlated. In the overdominant model of inheritance, there were not any association between SNPs and risk of OSCC. The overdominant model of inheritance is an attractive model since a single gene can potentially create the heterotic effect, but only a few such loci have been identified. Our finding in general may be an indicator for a higher susceptibility to cancer development not for a specific cancer.

The important roles of lncRNAs in proliferation, cell cycle regulation, apoptosis, and differentiation have been reported in molecular studies [13]. The expression level, structure, and stability of lncRNA may change by genetic mutation on lncRNA [14], thereby contributing to the pathogenesis of many disorders. For instance, it was shown that the lncRNA level was reduced by allelic changes in rs11752942 through binding micro-RNA, leading to increased esophageal squamous cell proliferation [15]. Different genetic variants of *H19* are able to regulate the aberrant expressions of *H19* and influence the activity of regulatory factors [16]. Many previous studies have corroborated the significant role of *H19* as an oncogenic molecule in different cancer cells and in tumorigenesis. Chen et al. in 2016 suggested that *H19* might increase gastric cancer cell invasion and migration [17]. Similar findings have been observed in the carcinoma cells of esophageal squamous cell [18, 19]. It was demonstrated that inhibiting the expression of *H19* in hepatocellular carcinoma cell lines reduced the ability of carcinoma cells to migrate and invade [20]. *LncRNA H19* is capable of increasing the metastasis of pancreatic cancer tissues through highly expressing pancreatic cancer tissues [21].

lncRNA SNPs can further affect the occurrence of tumors as well as the expression and function of genes [20]. Changes in lncRNA polymorphic site may influence its expression level and stability, hence affecting the development of tumors. lncRNA SNPs can influence lncRNA-miRNA interaction and modify and change the stability of lncRNA structures [22]. To date, various studies have shown the association between *H19* polymorphisms and several types of cancer. In a meta-analysis, Chu et al. reported the association between three *H19* polymorphisms (rs2839698, rs217727, and rs2107425) and cancer susceptibility [16]. It was also shown that the polymorphic site of rs217727 on *H19* was associated with the risk of breast cancer (OR = 0.79; 95%CI = 0.55 − 0.97) [23]. In a meta-analysis, the relationship between cancer susceptibility and *lncRNA H19* polymorphisms was studied, where rs2839698 G>A polymorphism was associated with digestive cancer risk; moreover, in the Caucasian populations, T allele variant of the rs2107425 C>T polymorphism had a protective effect against cancer progression [24]. It was demonstrated in another study that the rs217727 polymorphic site on *H19* was associated with a certain cancer (OR = 1.31, 95%CI = 1.03 − 1.67) [11]. In European Caucasians, *H19* genetic polymorphisms (rs2107425 and rs2839698) might also be related to the susceptibility of cancer [25]. Yin et al. found that homozygous variant genotype of rs2107425 might increase the risk of lung cancer [26]. Another study suggested that in urothelial cell carcinoma individuals, both *H19* SNPs rs2107425 and rs217727 polymorphic variants were susceptible to increased risks of muscle invasive tumors [27]. Wu et al. observed that an upstream SNP of *H19*, rs2107425, was associated with a lower risk of hepatocellular carcinoma [28].

The present study specified whether the SNP in the *lncRNA H19* gene is associated with OSCC in Iranian individuals by the use of tetra-primer ARMS-PCR which is an accurate, reliable, and simple method for genotyping single-nucleotide polymorphisms. This method includes a PCR reaction in a vial with two pairs of primers followed by electrophoresis on agarose gel [29]. Noteworthy, although previous studies have reported the role of *H19* in various diseases, the present research is the first to investigate the genetic variation of *H19* in association with OSCC in an Iranian population. The controls and cases were similar in baseline characteristic distributions and matched with age. The limitations of the present study were the different ethnic groups living in the southwest of Iran, the low sample size which might have affected the results, and environmental factors. More promising results would be observed with larger sample sizes, validating whether *H19* SNP could affect *H19* expression and OSCC etiology.

## 5. Conclusions

The present population-based case-control study provided the first evidence that *H19* rs217727 polymorphism might affect the risk of developing OSCC in an Iranian population. However, it is more preferable to use different ethnicities and a larger sample size to verify the results and perform function tests to reveal specific mechanisms.

## Figures and Tables

**Table 1:** Demographic characteristics of the sample.

Characteristics	Control (%)	Case (%)
Number	200 (100)	200 (100)
Males	156 (78)	156 (78)
Females	44 (22)	44 (22)
Age	55.21 ± 9.45	59.69 ± 16.24

**Table 2:** Patient inclusion and exclusion criteria.

Criteria	Description
Inclusion	(i) Histologically proven squamous cell carcinoma of the oral cavity (ii) Resectable tumor stage III or IV (iii) No tumor-specific pretreatment (iv) Informed consent

Exclusion	(i) Distant metastasis (ii) Secondary malignancies (iii) Pregnancy (iv) No disposing capacity or expected insufficient compliance

**Table 3:** The primers used for detection of rs217727 and rs2107425 polymorphisms in *lncRNA H19* gene.

Gene polymorphisms	Chr:position	Product size	Primers	Sequence (5′ to 3′)
rs217727	Chr11:1995678	Outer primers: 397 bp	FO	ATGACTCAGGAATCGGCTCTGGAAGGTG
			RO	GGGGAAACAGAGTCGTGGAGGCTTTGA
		C allele: 248 bp	FI	TCATCTTCATGGCCACCCCCTGCTGT
		T allele: 200 bp	RI	ATATGGTGGCTGGTGGTCAACCGTACG

rs2107425	Chr11:1999845	Outer primers: 266 bp	FO	ACTTGAGTCCCAGGCCATGACACTGAAG
			RO	CGGAATTGGTTGTAGTTGTGGAATCGGA
		T allele: 195 bp	FI	CGACCTGAAGATCTGGTGCGGCTACT
		C allele: 124 bp	RI	GGGTCATCTGGGAATAGGACACTCCTG

**Table 4:** The alleles and genotypes frequency of the *H19* polymorphisms in OSCC cases and healthy Controls.

Polymorphisms	Alleles *n* (%)		*χ* ^2^	*P* value	Genotypes *n* (%)			*χ* ^2^	*P* value	*P* value of HWE^a^
rs217727	C	T			CC	CT	TT			
Case	295 (73.75)	105 (26.25)	8.96	0.002	110 (55)	75 (37.5)	15 (7.5)	11.05	0.004	0.65
Control	330 (82.5)	70 (17.5)			133 (66.5)	64 (32)	3 (1.5)			
rs2107425										
Case	252 (63)	148 (37)	0.04	0.8	79 (39.5)	94 (47)	27 (13.5)	0.49	0.78	0.90
Control	249 (62.25)	151 (37.75)			74 (37)	101 (50.5)	25 (12.5)			

HWE, Hardy–Weinberg equilibrium.

**Table 5:** The association of *H19* polymorphism and OSCC susceptibility.

	Inheritance model	Genotype	Cases *n* (%) *n* = 200	Controls *n* (%) *n* = 200	OR (95% CI)	*P* value
rs217727	Codominant^a^	CC	110 (55)	133 (66.5)		
		CT	75 (37.5)	64 (32)	1.47 (0.93-2.15)	0.10
		TT	15 (7.5)	3 (1.5)	6.04 (1.70-21.42)	0.001
	Dominant^b^	CC	110 (55)	133 (66.5)		
		CT+TT	90 (45)	67 (33.5)	1.62 (1.08-2.43)	0.01
	Recessive^c^	CC+CT	185 (92.5)	197 (98.5)		
		TT	15 (7.5)	3 (1.5)	5.32 (1.51-18.69)	0.003
	Overdominant^d^	CC+TT	125 (62.5)	136 (68)		
		CT	75 (37.5)	64 (32)	1.27 (0.84-1.92)	0.24

rs2107425	Codominant	CC	79 (39.5)	74 (37)		
		CT	94 (47)	101 (50.5)	0.87 (0.57-1.33)	0.52
		TT	27 (13.5)	25 (12.5)	1.01 (0.53-1.89)	0.97
	Dominant	CC	79 (39.5)	74 (37)		
		CT+TT	121 (60.5)	126 (63)	0.89 (0.60-1.34)	0.60
	Recessive	CC+CT	173 (86.5)	175 (87.5)		
		TT	27 (13.5)	25 (12.5)	1.09 (0.60-1.95)	0.76
	Overdominant	CC+TT	106 (53)	99 (49.5)		
		CT	94 (47)	101 (50.5)	0.86 (0.58-1.28)	0.48

OR, odd ratio; CI, confidence interval. ^a^Codominant, major allele homozygotes vs. heterozygotes. ^b^Dominant, major allele homozygotes vs. heterozygotes + minor allele homozygotes. ^c^Recessive, major allele homozygotes + heterozygotes vs. minor allele homozygotes. ^d^Overdominant, major allele homozygotes + minor allele homozygotes vs. heterozygotes.

## Data Availability

No data were used to support this study.
